# Supplementary material for: Prevalence and predictors of Biker’s hand syndrome among the professional bike riders in Bangladesh: A cross-sectional study
Source: PLoS One. 2025 Sep 23;20(9):e0332228. doi: 10.1371/journal.pone.0332228 (PMC12456777; doi:10.1371/journal.pone.0332228)
Supplement: S2 Text — (DOCX) [file pone.0332228.s002.docx]

**Prevalence and predictors of Biker’s hand syndrome among the professional bike riders in Bangladesh**

Id No:

Name (optional):

Contact No:

| Q. N | Question | Answer | | |
| --- | --- | --- | --- | --- |
| **Section: A Demographic information** | | | | |
| 1 | Age |  | | |
| 2 | Gender | Male  Female | | |
| 3 | Self-reported weight |  | | |
| 4 | Self-reported height |  | | |
| 5 | BMI |  | | |
| 6 | Educational Qualification | SSC or bellow  HSC  Under-graduate  Post-graduate | | |
| 7 | Marital status | Married  Unmarried/Divorced/Widow | | |
| 8 | Religion | Muslim  Hindu  Others | | |
| 9 | Smoking habit | Yes  No | | |
| 10 | Alcohol habit | Yes  No | | |
| **Section: B work-related factors** | | | | |
| 11 | Riding status | Professional  Occasional | | |
| 12 | Average riding time (hours/per-day) | <6 hours  6-8 hours  >8 hours | | |
| 13 | How long have you been riding a bike | 0-5 years  6-10 years  >10 years | | |
| 14 | How much is your monthly income? | ≤30000 TK  31000-50000 TK  >50000 TK | | |
| 15 | Is the hand grip for your bike comfortable for you | Yes  No | | |
| 16 | Do you think your bike fits your body physique? | Yes  No | | |
| 17 | Do you use any safety equipment (gloves/wrist band) while riding the bike? | Yes  No | | |
| 18 | Bike engine capacity | ≤150 cc | | |
|  |  | >150 cc | | |
| 19 | Do you have pain, ache or discomfort in the following shaded area while bike riding?  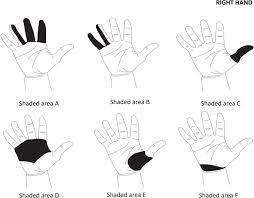 | Area | Rt | Lt |
|  |  | No pain |  |  |
|  |  | Area A |  |  |
|  |  | Area B |  |  |
|  |  | Area C |  |  |
|  |  | Area D |  |  |
|  |  | Area E |  |  |
|  |  | Area F |  |  |
|  |  |  | | |
| 19 | During the last work week how often did you experience ache, pain and discomfort in your hands | Never | | |
|  |  | 1-2 times | | |
|  |  | 3-4 times | | |
|  |  | Once every day | | |
|  |  | Multiple times | | |
|  | a. If you experienced ache, pain discomfort, how uncomfortable was this? | Slightly uncomfortable | | |
|  |  | Moderately uncomfortable | | |
|  |  | Very uncomfortable | | |
|  | b. If you experienced ache, pain discomfort, did this interfere your work? | Not at all | | |
|  |  | Slightly interfered | | |
|  |  | Substantially interfered | | |
