# Supplementary material for: Prevalence and predictors of Biker’s hand syndrome among the professional bike riders in Bangladesh: A cross-sectional study
Source: PLoS One. 2025 Sep 23;20(9):e0332228. doi: 10.1371/journal.pone.0332228 (PMC12456777; doi:10.1371/journal.pone.0332228)
Supplement: Attachment — Submitted filename: Response to Reviewers.docx [file pone.0332228.s006.docx]

**Reviewer #1**

This cross-sectional study investigates the prevalence and predictors of Biker’s Hand Syndrome (BHS) among 630 professional ride-share motorcyclists in Bangladesh. Using the Cornell Hand Discomfort Questionnaire, it reports a high prevalence of hand discomfort (58.3% right, 51.3% left) and identifies significant associations with age, BMI, years of riding, engine capacity, grip suitability, and bike-body fit. The study is methodologically sound, with appropriate sampling and statistical modeling. It addresses a research gap by focusing on hand-specific musculoskeletal disorders in a developing country occupational setting. Its practical implications are clear—highlighting ergonomic risk factors that could inform health policy, rider training, and bike design. However, the reliance on self-reported symptoms without clinical validation limits diagnostic certainty, and factors like comorbidities or road conditions were not explored. Despite these limitations, the findings offer valuable direction for ergonomic interventions and occupational health strategies in the ride-sharing sector.

**Reviewer’s comment:** 1. Lack of Clinical Validation of Biker’s Hand Syndrome: While the authors acknowledge using a self-reported questionnaire, they do not explicitly discuss the limitation of lacking clinical or neurological confirmation (e.g., no physical or diagnostic assessment to differentiate BHS from other hand disorders). This remains a significant methodological limitation.

**Reply to the reviewer’s comment:** Thank you for your valuable comment and for highlighting this important issue. We have now addressed it in the discussion section as a limitation in the revised text.

**Reviewer’s comment:** 2. Omission of Confounding Variables: The study does not mention or control for comorbidities such as diabetes, arthritis, or history of repetitive hand use, which could influence symptom reporting. These are important omissions that were not addressed in the limitations.

**Reply to the reviewer’s comment:** Thank you for your insightful remark and for emphasizing this significant matter. The issue has been resolved in the discussion section as a limitation in the amended text.

**Reviewer’s comment:** 3. No Justification for Right vs. Left Hand Modeling Strategy: Although right- and left-hand data are reported separately, the rationale for modeling them independently is not discussed, and inter-hand correlation is not addressed. This may affect interpretation of results.

**Reply to the reviewer’s comment:** Thank you for bringing up this issue. We aim to investigate the prevalence and predictors of hand syndrome among professional ride-sharing riders in Bangladesh. When riding a motorcycle, the right and left hands engage differently with the clutch, throttle, and hand brakes, necessitating the use of both hands. For that reason, we are modeling them independently. For your clarification, we now added the following text in the revised manuscript: ‘’The highest prevalence of pain in the right hand was reported in area F (the palm and near the wrist), which may be linked to prolonged pressure on the handlebar while using the throttle. In contrast, a significant prevalence of pain was observed in area D of the left hand, likely associated with repetitive use of the clutch. These findings highlight the importance of motorcycle handlebar ergonomics.’’

**Reviewer’s comment:** 1. Terminology and Clarity: “Biker’s Hand Syndrome” is treated as a distinct condition without formal clinical recognition. A brief clarification of the term’s novelty or how it relates to established conditions (e.g., hand-arm vibration syndrome) would improve clarity.

**Reply to the reviewer’s comment:** Thank you for your valuable comment, we already defined the terminology in the introduction section of the manuscript as follows: "Biker's hand syndrome is a condition characterized by pain, discomfort, paresthesia, or numbness in the hands. The symptoms result from excessive usage, nerve compression, or excessive pressure on the hand during prolonged durations of bike riding.’’

**Reviewer’s comment:** 2. Formatting and Typographical Issues: Minor inconsistencies remain (e.g., “echo friendly” instead of “eco-friendly”), but these are easily corrected.

**Reply to the reviewer’s comment:** Thank you, corrected in the revised text.

**Reviewer’s comment:** 3. Limited Depth on Preventive Strategies: Although prevention is mentioned (e.g., bike fit, stretching), these suggestions are not supported with specific references or actionable guidelines, making the recommendations feel superficial.

**Reply to the reviewer’s comment:** Thank you for your valuable comment. We now addressed the following statements in the revised text: ‘’Bikers should use comfortable bike fitted according to their body physique and should use comfortable hand gripper to avoid pain. Individuals whose motorcycles do not match their body physique have more than five times the likelihood of experiencing hand discomfort. It is strongly advised that ride-sharing platforms implement policies for motorcycle selection or adjustments based on the rider’s height and weight.’’

**Reviewer’s comment:** 4. Lack of Visual Summary: A flowchart of the sampling and data analysis procedure or a forest plot summarizing regression findings would improve accessibility, especially for visual readers.

**Reply to the reviewer’s comment:** Thank you for your valuable suggestion. The revised text now includes Figure 2, which provides details on the sampling and data analysis.

**Reviewer’s comment:** 5. CHDQ Validation Context: The CHDQ is cited, but its cultural adaptation and validation for Bangladeshi riders is not explained. This should be clarified to support its use in this specific context.

**Reply to the reviewer’s comment:** The cross-cultural adaptation or language validation for this questionnaire in Bangla has not yet been conducted. However, to ensure consistency, we employed forward and backward translation for the questionnaire, and we trained all data collectors before the data collection process.

**Reviewer’s comment:** 1. Line number: Please add line number to help the reviewer to read and locate the issues

**Reply to the reviewer’s comment:** added in the revised text.

**Reviewer’s comment:** echo friendly (pdf p.15, article p.9, Survey administration section): should be eco-friendly

**Reply to the reviewer’s comment:** Thank you for highlighting our typographical mistake. Corrected in the revised text.

**Reviewer’s comment:** hand gripper that provides comfort (abstract and conclusion): it would be better “comfortable hand gripper”

**Reply to the reviewer’s comment:** Thank you for your comment. Corrected in the revised text.

**Reviewer’s comment:** MBI (pdf p.20, article p.14, Discussion): BMI

**Reply to the reviewer’s comment:** Thank you for highlighting our typographical mistake. Corrected in the revised text.

**Reviewer’s comment:** affirmative or negative response (pdf p. 12, article p.6, Ethical consideration section): it would be better “consent or decline”

**Reply to the reviewer’s comment:** Corrected in the revised text.

**Reviewer #2**

The manuscript presents a timely and relevant study on the prevalence and predictors of biker’s hand syndrome among professional motorcycle riders in Bangladesh—a growing occupational group facing significant musculoskeletal challenges. The topic is important from both public health and ergonomic perspectives, particularly in low- and middle-income countries where ride-sharing services are expanding rapidly. The study design, data collection methods, and statistical analyses are generally sound, and the findings offer valuable insights into a largely understudied occupational health issue. However, several revisions are needed to improve clarity, methodological transparency, and scientific rigor before the manuscript can be considered for publication.

**Reviewer’s comment:** The Introduction effectively establishes the context of motorcycle use in Bangladesh and defines biker’s hand syndrome with reference to pain, discomfort, and functional impact. The justification for the study is reasonable, highlighting a gap in the literature regarding professional riders. However, the claim that “no study has yet been conducted on this serious issue” is likely an overstatement. While the specific focus on ride-sharing riders in Bangladesh may be novel, similar conditions have been studied in cyclists and motorcyclists elsewhere. A more precise statement—such as “no prior study has specifically investigated hand discomfort among professional ride-sharing riders in Bangladesh”—would be more accurate and defensible. Additionally, the introduction would benefit from a clearer conceptual framework linking individual, occupational, and environmental factors to the development of hand syndrome.

**Reply to the reviewer’s comment:** Thank you for your comments. As per your recommendation we now replace the statement “no study has yet been conducted on this serious issue” with “no prior study has specifically investigated hand discomfort among professional ride-sharing riders in Bangladesh” in the revised text. We also included a conceptual framework in the revised version of the manuscript.

**Reviewer’s comment:** The Methods section is generally well-structured, but several improvements are needed. First, the definition of “professional biker” requires clarification. Does this term refer exclusively to ride-sharing drivers (e.g., Pathao, Uber), or does it include other types of motorcycle-based workers? A clear operational definition (e.g., income source, daily riding hours) should be provided. Second, while the use of two-stage cluster sampling is appropriate, the process of randomization is not sufficiently detailed. Please specify how clusters (e.g., bus stations, markets) were selected—was a sampling frame used, or was selection based on convenience? Third, although the Cornell Hand Discomfort Questionnaire (CHDQ) was used, only the presence/absence of pain was reported, not severity or frequency. This limits the depth of analysis. Please justify this decision and clarify whether the full CHDQ was administered. Additionally, while back-translation was performed, there is no mention of pilot testing results or reliability assessment (e.g., Cronbach’s alpha) in the target population, which is essential for validating translated instruments.

**Reply to the reviewer’s comment:** Thank you for your valuable comments. The revised manuscript includes the following statement to clarify who is a professional biker:

The term "professional biker" specifically refers to ride-sharing drivers (such as Pathao and Uber) who generate income by providing ride-sharing services and who ride bikes for at least 3 to 4 hours each day. The cluster sampling process is now described in the revised text.

Thirdly, the objective of this study was to determine the prevalence and predictors of biker's hand syndrome among professional bike riders in Bangladesh. We reported the frequency, severity, and interference in Table 1; however, in line with our objective, we focused solely on the presence or absence of pain (prevalence) in the shaded area and analyzed the data accordingly.

To ensure data collectors were competent and to identify any inconsistencies during data collection, we conducted a pilot test of the questionnaire with five participants for each data collector. We acknowledge a limitation in that we did not assess the reliability of the pilot study, as noted in the limitations section of the revised manuscript.

**Reviewer’s comment:** The Results section is comprehensive and well-presented, with clear reporting of descriptive statistics, prevalence rates, bivariate associations, and multivariable regression models. The separate analysis of right- and left-hand symptoms is clinically meaningful. However, several issues require attention. First, the dependent variable in the logistic regression— “presence of pain in any of six hand regions”—should be explicitly defined in the text or in a table footnote. Second, while adjusted odds ratios (aOR) and 95% CIs are reported, p-values for each predictor are missing. These should be included for full transparency. Third, model fit statistics (e.g., Hosmer-Lemeshow test result, p-value) and multicollinearity diagnostics (e.g., VIF values) should be reported to support the validity of the regression model. Fourth, the finding that normal BMI is associated with higher odds of hand syndrome compared to obesity is counterintuitive and requires careful interpretation in the Discussion—possibly due to confounding or differential exposure patterns.

**Reply to the reviewer’s comment:** Thank you for your comment. The dependent variable, which refers to the presence or absence of pain in the six shaded areas, is already included in Table 1. The p-value is mentioned in Table 4; however, we have also included it in the text of the revised manuscript. The model fitness statistics also added in the data analysis section.

**Reviewer’s comment:** The Strengths, Limitations, and Future Recommendations section acknowledges key limitations, including the cross-sectional design and inability to infer causality, which is commendable. However, the claim that the findings are generalizable to the “entire nation’s populace” is inaccurate and should be revised. The study was conducted in only two metropolitan areas and among a specific occupational group, limiting broader applicability. Instead, the authors should emphasize urban generalizability and suggest future studies in rural or semi-urban settings. The omission of road surface quality and traffic conditions as measured variables is a valid limitation, as these factors significantly influence hand vibration and strain. Future studies should consider incorporating objective or self-reported measures of these environmental exposures.

**Reply to the reviewer’s comment:** We now corrected the generalizability statement as follow in the revised text: ‘’Employing a random sample method from two separate metropolitan areas improves result precision and reduces selection bias. Encompassing the entire nation's populace could increase the generalizability of the findings.’’

**Reviewer’s comment:** Finally, the future research recommendations are somewhat generic. To enhance impact, they should be more specific and actionable—such as recommending longitudinal designs to assess symptom progression, or intervention studies evaluating ergonomic modifications (e.g., handlebar design, glove use). Comparative studies with non-professional riders or other transport workers could also deepen understanding of occupational risk factors.

**Reply to the reviewer’s comment:** Thank you for your insightful comment. The future recommendation section is now upgraded, as per your suggestion in the revised text.

**Reviewer’s comment:** Minor issues include repetitive phrasing, inconsistent use of terms (e.g., “pain, ache, or discomfort”), and lack of a figure showing the CHDQ hand map (regions A–F). Including such a figure would greatly improve clarity. Additionally, ensure all statistical software (e.g., SPSS, R) and version numbers are reported.

**Reply to the reviewer’s comment:** We already included figure 1, which includes a hand map diagram (regions A-F). We also reported the statistical software name and version in the revised text.

**Reviewer’s comment:** In summary, this is a valuable contribution to occupational health research with strong potential. With the suggested revisions—particularly in clarifying methods, improving statistical reporting, refining interpretations, and enhancing future directions—the manuscript will be well-suited for publication in an international journal.

**Abstract**

The abstract is well-structured and communicates key findings effectively. However, with the suggested revisions—particularly in enhancing methodological transparency, statistical reporting, interpretative clarity, and strategic use of keywords—it can become a stronger, more impactful representation of the study, significantly improving its chances of acceptance and readership in a high-quality journal.

**Reviewer’s comment:** The background section provides a general introduction to the topic but could be strengthened with more specific contextual details. The phrase "gaining popularity" is too vague; it would be more impactful to explicitly mention the rapid growth of ride-sharing platforms in Bangladesh and the increasing economic reliance on this occupation. Additionally, the term "biker’s hand syndrome" is used without a brief definition, which may be unclear to non-specialist readers. It is recommended to define the condition concisely—such as "an ergonomic disorder caused by prolonged pressure on the ulnar nerve and hand tissues during motorcycle riding." These improvements would enhance clarity, relevance, and professionalism, ensuring that the scientific and public health significance of the study is immediately apparent to the reader.

**Reply to the reviewer’s comment:** Thank you for your valuable comment, which helps us improve the quality of the manuscript. In response to your comment, we have now corrected the issue in the revised text.

**Reviewer’s comment:** While the Methods section includes essential elements such as study design, sample size, measurement tool (CHDQ), and statistical analysis, it omits several critical details necessary for assessing methodological rigor. For instance, the cities studied (Dhaka and Sylhet), the sampling technique (two-stage cluster sampling), and the data collection method (face-to-face interviewer-administered questionnaire) are not mentioned. Including these details would significantly improve transparency and allow readers to better evaluate the study’s validity and reproducibility. These elements reflect a systematic and well-controlled approach, and their omission weakens the perceived quality of the research. It is strongly recommended to briefly incorporate this information into the abstract to strengthen the methodological credibility of the study.

**Reply to the reviewer’s comment:** Thank you for bringing this issue to our attention. We have now incorporated those statements into the revised text, following your feedback.

**Reviewer’s comment:** The reporting of hand discomfort prevalence (58.3% in the right hand, 51.3% in the left) is appropriate and informative. However, the interpretation of predictors lacks sufficient statistical clarity. The statement that "individuals aged 31–40 had a lower prevalence" is ambiguous without specifying the reference group (i.e., those over 40 years), which may lead to misinterpretation. Similarly, the claim that "normal-weight and underweight riders had higher odds" is potentially misleading, as it combines two distinct BMI categories without clarifying their individual effects. Moreover, the absence of adjusted odds ratios (aOR) and 95% confidence intervals (CI) undermines the scientific rigor of the abstract. Including these values—even in abbreviated form—would allow readers to assess the strength and precision of the reported associations and significantly enhance the credibility of the findings.

**Reply to the reviewer’s comment:** Thank you for your comment. We excluded the adjusted odds ratios (aOR) and 95% confidence intervals (CI) from the abstract due to word limitations. However, following your recommendation, we have now included them in the revised text.

**Reviewer’s comment:** The current conclusion offers general recommendations such as "performing stretching exercises" and "using a comfortable handle grip," but these are not explicitly linked to the study’s key findings—such as poor bike-to-body fit or low riding experience. To increase impact, the conclusion should directly reflect the strongest results, for example: "Improving motorcycle-to-body fit could reduce hand syndrome risk by over fivefold." Additionally, recommendations should extend beyond individual-level actions to include systemic interventions involving ride-sharing companies, occupational health programs, and policymakers. Using stronger language—such as "should be implemented" instead of "it is advisable"—would make the conclusions more authoritative and compelling. A more targeted, policy-relevant conclusion would better reflect the public health significance of the findings.

**Reply to the reviewer’s comment:** Thank you for bringing this issue to our attention. We have now enhanced the conclusion in accordance with your recommendation in the revised text.

**Reviewer’s comment:** The current keywords are relevant but can be improved. First, they should be listed in alphabetical order to appear more professional. Second, adding terms such as "occupational health" or "ergonomics" would enhance the article’s visibility in scientific databases and align it with broader research domains. These terms help categorize the study within established public health and safety frameworks. Additionally, while "biker’s hand syndrome" is a novel and descriptive term, it should be supported by more standard keywords like "musculoskeletal disorders" or "hand discomfort" to improve searchability and indexing. Optimizing the keywords will increase the article’s discoverability and ensure it reaches a wider, interdisciplinary audience.

**Reply to the reviewer’s comment:** Thank you for your feedback. We have removed the keyword "bikers hand syndrome", substituted it with "musculoskeletal disorders", and added "occupational health" in the revised text.

**Reviewer’s comment:** The choice of keywords would benefit from alignment with the official Medical Subject Headings (MESH) terminology maintained by the U.S. National Library of Medicine. Using standardized MESH terms enhances indexing accuracy, improves discoverability in databases such as PubMed/Medline, and increases the article’s visibility to the global research community.

**Reply to the reviewer’s comment:** Thank you for your comment; we have included the keywords from the Medical Subject Headings (MESH) terminology that is maintained by the U.S. National Library of Medicine.

**Introduction**

This introduction has a suitable structure, a clear definition of biker’s hand syndrome, and a general justification for the study. However, with the implementation of these revisions—including an earlier focus on the study population, the use of more relevant sources, a refined innovation claim, and the addition of a risk factor framework—it can become a strong, coherent, and impactful introduction that enhances the likelihood of acceptance in international journals.

**Reviewer’s comment:** The introduction begins with general commentary on the popularity of motorcycles in Asian countries, but it does not narrow its focus to the specific study population—professional ride-sharing motorcycle riders in Bangladesh—until the end of the section. This broad and indirect approach may dilute the clarity and impact of the introduction. To enhance relevance and reader engagement, it would be more effective to focus from the outset on the occupational riders in Bangladesh, who are exposed to unique stressors such as prolonged working hours, handlebar vibration, and poor ergonomic conditions. Rewriting the introduction to center directly on this group can help establish the study’s social and occupational significance early on and strengthen the logical connection between the problem and the target population.

**Reply to the reviewer’s comment:** Thank you for your valuable suggestion, which has greatly assisted us in improving our manuscript. We have now refined the introduction to concentrate specifically on the ride-sharing population in Bangladesh. Additionally, we have incorporated several further stressors associated with ride-sharing in the revised manuscript.

**Reviewer’s comment:** While prior research on cycling (e.g., mountain biking and road cycling) may offer some insights into hand discomfort, key distinctions between bicycles and motorcycles are overlooked in the introduction. Motorcycles are heavier, produce more vibration, operate at higher speeds, and are often used for longer periods—factors that can potentially exacerbate hand-related syndromes. Citing bicycle-based studies without acknowledging these critical differences may lead to biased or inappropriate generalizations. The introduction would benefit from clarifying these distinctions and then using bicycle studies as a conceptual starting point to justify exploring this issue in motorcyclists.

**Reply to the reviewer’s comment:** Thank you for your comment, we now added the following statement in the revised text: “Although motorcycles are heavier, produce more vibration, operate at higher speeds, and are often used for longer periods than mountain biking and road cycling, these symptoms are prevalent in mountain biking and road cycling, as shown in prior research.”

**Reviewer’s comment:** The epidemiological and social burden of the problem also requires strengthening. Although the introduction mentions consequences such as reduced productivity, absenteeism, and strain on the healthcare system, these claims lack supporting quantitative data or region-specific statistics. Incorporating findings from neighboring South Asian countries (e.g., India, Indonesia, or Pakistan) on the prevalence or economic impact of hand discomfort among professional riders would enhance the justification for this study. This contextual information would help readers grasp the population-level significance and policy relevance of the issue.

**Reply to the reviewer’s comment:** Thank you for your comment; we now included the following statement with reference to strengthen the study: Hand/Arm symptoms cause productivity loss, which includes 11% sickness absence and 36% productivity loss [15].

**Reviewer’s comment:** The phrase, *"To the best of our knowledge, no study has yet been conducted on this serious issue,"* may come across as an overstatement. It is likely that studies on hand discomfort in similar occupational groups exist, even if not specifically targeting ride-sharing motorcycle riders in Bangladesh. This assertion may be challenged by reviewers. A more cautious and accurate wording is recommended, such as: *"To the best of our knowledge, no prior study has specifically investigated the prevalence and predictors of hand discomfort among professional ride-sharing motorcycle riders in the context of Bangladesh."*

**Reply to the reviewer’s comment:** Thank you for your valuable suggestion; we have now modified our text accordingly.

**Reviewer’s comment:** This version avoids exaggeration while still emphasizing the novelty of the study. ،The lack of a conceptual framework or risk factor model is noticeable. The introduction would be strengthened by a brief statement outlining how various factors—individual (e.g., age, BMI), occupational (e.g., riding hours, handlebar design), and biomechanical (e.g., vibration, grip force)—may interact to contribute to hand discomfort. For example: *"Risk factors may include individual characteristics (e.g., age, BMI), occupational exposures (riding duration, handlebar design), and biomechanical stressors (vibration, sustained grip force), which collectively contribute to the development of hand symptoms."* This approach not only enhances conceptual clarity but also provides a theoretical basis for the study’s variable selection.

**Reply to the reviewer’s comment:** Thank you for your valuable suggestion; we have now modified our text accordingly.

**Reviewer’s comment:** The concluding sentence of the introduction— *“This study aimed to find out the prevalence and predictors of biker hand syndrome…”*—is clear and appropriately worded. However, it could be made more impactful and scholarly by emphasizing both its practical importance and preventive potential. For example: *"Therefore, this study aims to determine the prevalence of biker’s hand syndrome and identify modifiable risk factors among professional ride-sharing motorcycle riders in Bangladesh, with the goal of informing preventive occupational health interventions."*

**Reply to the reviewer’s comment:** Thank you for your valuable suggestion; we have now modified our text accordingly.

**Methodology**

This study has a solid methodological foundation. With the implementation of these revisions—particularly in clarifying sampling procedures, reporting instrument validation, explaining pilot results, managing missing data, specifying measurement scales, naming statistical software, and improving language clarity—the manuscript can achieve a higher standard of scientific rigor and suitability for publication in international journals.

**Reviewer’s comment:** The present study was conducted as cross-sectional research in the urban areas of Dhaka and Sylhet, Bangladesh, from January to June 2024, which is appropriate for assessing the prevalence and associated factors of hand discomfort among motorcycle riders. However, the definition of “occupational riders” requires greater precision, as the absence of a clear operational definition (e.g., minimum daily riding hours or proportion of income derived from riding) may introduce selection bias and affect the validity of results. Additionally, the rationale for selecting only two cities without demographic, economic, or geographic justification may lead to sampling bias and should be clarified. Furthermore, the name of the primary host university or research institution should be explicitly stated to enhance the study’s institutional credibility.

**Reply to the reviewer’s comment:** Thank you for bringing this issue to our attention and allowing us the chance to enhance the quality of the manuscript. At present, Uber operates in twenty major cities across Bangladesh; however, for the sake of the researcher's convenience, we have included participants from the Dhaka and Sylhet districts. We also included the definition of an 'occupational rider' in the first paragraph of the methodology section in the revised text.

**Reviewer’s comment:** Ethical approval for this study was obtained from the Ethical Review Board of the Institute of Physiotherapy, Rehabilitation, and Research (BPA-IPRR/IRB/07/12/2023/119), and adherence to the ethical principles of the Declaration of Helsinki (2013) and the Bangladesh Medical Research Council guidelines is highly commendable. The use of digital informed consent via Google Forms is a modern and acceptable approach. However, it should be clearly emphasized that participants were given sufficient time to review the study information and ask questions before consenting. Moreover, the lack of mention regarding participants’ right to withdraw from the study at any time without penalty, as well as the absence of details on secure and confidential data storage (e.g., encryption, restricted access), represents a gap in the ethical section that should be addressed.

**Reply to the reviewer’s comment:** Thank you for highlighting this issue. Prior to participating in the study, the participants had sufficient time to review the study information and ask questions regarding it before consenting. We included the following statement in the revised text: ‘We assure participants that they may withdraw from the study at any time without penalty. Furthermore, their information will be kept confidential and will only be used for publication in the journal.’

**Reviewer’s comment:** The study population was defined as professional motorcycle riders using ride-sharing platforms such as Uber and Pathao, which aligns well with the research objective. However, the distinction between “professional” and “occasional” riders is not explained in this section, despite the variable being used later in the analysis. Without a precise operational definition (e.g., individuals who ride more than 6 hours per day or derive over 70% of their income from riding), misclassification of participants is possible. Therefore, it is recommended to provide a clear and explicit definition of “occupational riders” in the methods section to improve methodological transparency.

**Reply to the reviewer’s comment:** Thank you for bringing this important issue to our attention. We acknowledge our oversight in not addressing it previously. To clarify, we have now incorporated the following statement in the revised text: ''either a professional who generates income solely from ride sharing or an occasional rider who shares rides for 3–4 hours to earn extra income alongside other work'' for the clarification.

**Reviewer’s comment:** The use of a two-stage cluster sampling method in public areas such as bus and train stations and markets is a suitable and methodologically sound approach for accessing a dispersed population of riders. The use of motorcycle registration numbers as unique identifiers is a smart strategy to prevent data duplication. However, the process of randomization in cluster selection requires greater clarity: was there a comprehensive list of locations, and were they selected via simple random sampling, or was the selection based on convenience? Additionally, the use of Google Forms for interviewer-administered data collection represents a good combination of technology and human interaction, and the training of interviewers by the principal investigator indicates high-quality control.

**Reply to the reviewer’s comment:** For cluster randomization, we began by listing the potential bus and train stations in Dhaka and Sylhet. In Dhaka, there are four intercity bus stations and two train stations, while Sylhet has one intercity train station and one bus stations. Additionally, there are numerous local bus stations and markets in Dhaka and Sylhet, which we also documented. Initially, we collected data from all the large bus and train stations; subsequently, we compiled a list of the local stations and markets and selected the twenty potential locations using lottery methods.

**Reviewer’s comment:** In the sampling section, the term “two-stage cluster sampling” is used, but the steps of this method are not described in sufficient detail. To enhance reproducibility and methodological transparency, the stages should be clearly outlined: in the first stage, public locations such as bus and train stations and markets were randomly selected (ideally from a comprehensive list or city map); in the second stage, motorcycle riders present at these locations during the research team’s visit were enrolled after eligibility screening and informed consent. Lack of clarity regarding the randomization process for selecting locations or individuals may lead to selection bias; therefore, it is recommended to revise and expand this section with precise details in the manuscript.

**Reply to the reviewer’s comment:** For cluster randomization, we began by listing the potential bus and train stations in Dhaka and Sylhet. In Dhaka, there are four intercity bus stations and two train stations, while Sylhet has one intercity train station and one bus stations. Additionally, there are numerous local bus stations and markets in Dhaka and Sylhet, which we also documented. Initially, we collected data from all the large bus and train stations; subsequently, we compiled a list of the local stations and markets and selected the twenty potential locations using lottery methods.

**Reviewer’s comment:** The inclusion criteria—aged 18–50 years, registered with a ride-sharing service, and willing to participate—are logical and clearly defined. The exclusion of food delivery riders is justifiable, as their hand usage patterns and repetitive load differ from passenger drivers and could act as a confounding variable. However, the six-month restriction for history of hand surgery or road traffic accidents requires justification, as some neurological or joint injuries may have longer-term effects. Additionally, the use of a pre-participation checklist is an appropriate measure to ensure accurate application of criteria, and it is recommended to include this checklist in the appendix or main text.

**Reply to the reviewer’s comment:** A "six-month" exclusion criterion serves to enhance the homogeneity of the study population by ensuring that participants either have a consistent baseline or have remained in a stable state for a minimum of six months. This approach aims to bolster the reliability and validity of the data, minimize the confounding effects of rapidly changing variables or recent interventions, increase the likelihood of identifying a clear treatment effect, and mitigate the risk of bias stemming from factors potentially linked to shorter durations. The checklist includes the exclusion criteria which is already mentioned in the text.

**Reviewer’s comment:** The sample size was correctly calculated using the standard formula for single population proportions (n = Z²p(1-p)/d²), with p=0.5 (most conservative estimate), d=0.04 (4% margin of error), and Z=1.96 (95% confidence level), resulting in a base sample of 600. Increasing this to 630 to account for 5% non-response or incomplete forms is a standard and logical adjustment. However, the reported actual non-response rate of 16% is higher than anticipated and requires explanation (e.g., reasons for refusal). Furthermore, the lack of detailed reference to source [15] for the sample size formula may raise questions; it is advisable to either fully describe the source or validate the formula with established statistical references.

**Reply to the reviewer’s comment:** Thank you for your valuable comment. The revised text addresses the causes of refusal related to insufficient time and a reluctance to engage. Additionally, we have replaced reference 15 with a more appropriate source in the updated text.

**Reviewer’s comment:** The study questionnaire includes demographic, occupational, and the standardized Cornell Hand Discomfort Questionnaire (CHDQ), which is content-appropriate. The use of back-translation for the Bengali version is a valid and essential method to preserve meaning and accuracy. The pilot test with five participants meets the minimum requirement, but it would be beneficial to report reliability metrics (e.g., Cronbach’s alpha) for the translated questionnaire to strengthen its validity. More importantly, the use of only the “presence/absence of pain” component of the CHDQ, while the full instrument includes severity, frequency, and interference with work, may result in loss of valuable information and requires justification.

**Reply to the reviewer’s comment:** Thank you for your valuable comment. The primary objective of this study was to determine the prevalence and predictors of biker's hand syndrome among professional bikers in Bangladesh. Consequently, we focused solely on reporting the presence or absence of pain. To ensure data collectors were competent and to identify any inconsistencies during data collection, we conducted a pilot test of the questionnaire with five participants for each data collector. We acknowledge a limitation in that we did not assess the reliability of the pilot study, as noted in the limitations section of the revised manuscript.

**Reviewer’s comment:** Although the validity of the CHDQ is cited from Dr. Oguzhan Erdinc’s study (2016), it is not clearly stated whether the instrument was previously validated in the population of motorcycle riders or within the Bangladeshi context. Given cultural differences, literacy levels, and variations in pain perception, the translated and adapted tool may require re-validation. Therefore, researchers should clarify whether the reliability of the instrument (e.g., via Cronbach’s alpha) was assessed in the target population or pilot group. Failure to report this may affect the credibility of the findings.

**Reply to the reviewer’s comment: To the best of our knowledge, no research has yet been conducted involving motorbikes to investigate hand syndrome using this questionnaire. One limitation of our study is that we did not evaluate the validity and reliability of the instrument. Nonetheless, we have acknowledged this limitation in the relevant section of the revised manuscript.**

**Reviewer’s comment:** While it is mentioned that the questionnaire was piloted with five participants, insufficient details are provided about the outcomes of this phase. For example, did participants experience confusion with any questions? Were any items ambiguous or double-barreled? Most importantly, were any changes made to the content, structure, or order of questions after the pilot? Reporting these details not only demonstrates methodological rigor but also helps readers better understand the quality of the measurement tool. Therefore, it is recommended to expand this section with a brief description of pilot findings and any corrective actions taken.

**Reply to the reviewer’s comment:** Our main goal for the pilot data collection was to assess the effectiveness of the data collector and to identify any potential confusion that participants might have had regarding the questions. The questionnaire worked well for both the data collector and the participants, and no modifications were required. We included the following statement in the revised text: ‘’The questionnaire worked well for both the data collector and the participants, and no modifications were required.’’

**Reviewer’s comment:** Questions such as “Is the handlebar comfortable for you?” and “Does the motorcycle fit your body physique?” require measurement clarity. It is unclear how these items were assessed: were they yes/no responses, a 5-point Likert scale (from “strongly disagree” to “strongly agree”), or another qualitative indicator? Lack of clarity regarding the response scale complicates data interpretation and limits comparability with other studies. Therefore, it is recommended to explicitly state the response format (qualitative or quantitative) and measurement scale for each question in the methods section.

**Reply to the reviewer’s comment:** The variable “Is the handlebar comfortable for you?” and “Does the motorcycle fit your body physique?” were assessed by yes/no responses. However, we now included the response format and measurement scale in the revised manuscript.

**Reviewer’s comment:** Independent variables were categorized into groups (e.g., age, BMI, education, income, riding hours), which is statistically appropriate. Converting the CHDQ outcome into a binary variable (pain present/absent) is reasonable for logistic regression analysis. However, the definition of “case” as pain in any of the six hand areas requires justification. Is this definition consistent with similar studies? Is pain in one area sufficient, or should at least two areas be involved? This should be clarified to ensure comparability with other research.

**Reply to the reviewer’s comment:** Pain in one region usually defines a case if it fits other criteria like duration and intensity. For example, The Nordic musculoskeletal disease questionnaire can be used in large-scale epidemiological studies and surveys to determine the prevalence of MSK pain in a community. To achieve relevant and comparable findings, a study's definition of "musculoskeletal pain" should contain criteria relating to duration, frequency, and functional effect. Pain in any one location of the body might serve as a basic screen.

**Reviewer’s comment:** Descriptive statistics (frequencies, percentages, means, standard deviations) are appropriately used to present the data. The chi-square test for examining associations between categorical variables and hand discomfort is suitable. The use of binary logistic regression to identify predictive factors, along with reporting odds ratios (OR) and 95% confidence intervals (CI), is a standard and robust approach. Model fitness assessed via the Hosmer-Lemeshow test and multicollinearity checked using VIF (≤5) indicate careful attention to model quality. However, the absence of information about the statistical software used (e.g., SPSS, R) and the method of variable entry into the model (e.g., Enter or Stepwise) is a limitation that should be corrected to ensure reproducibility.

**Reply to the reviewer’s comment:** Thank you for your valuable comment. Included in the revised text.

**Reviewer’s comment:** No information is provided on how missing or incomplete data were handled, despite its direct impact on analytical validity. For instance, if certain questions were left unanswered, were techniques such as listwise deletion, imputation, or sensitivity analysis applied? Although Google Forms typically prevents submission of incomplete forms, reducing the likelihood of missing data, this should still be explicitly stated. It is recommended that the authors clarify whether “all forms were complete with no missing data” or, if not, describe the method used for handling missing values.

**Reply to the reviewer’s comment:** Thank you for highlighting this crucial issue. We now included the statement ‘’all forms were complete with no missing data’’ in the first section of the results in the revised text.

**Reviewer’s comment:** Some sentences in the methods section are repetitive and suffer from weak grammatical structure. For example, the sentence “Data was collected via an interviewer administered questionnaire, which was then translated into Bangla and then translated back into English…” contains redundant use of “then” and awkward phrasing. Additionally, the description of the data collection process includes long, disorganized sentences lacking a logical sequence. This may hinder reader comprehension. Therefore, it is recommended to revise the text for clarity, conciseness, and minimal repetition. Using short sentences, active voice, and a clear chronological order (e.g., interviewer training, pilot testing, data collection) would significantly improve readability.

**Reply to the reviewer’s comment:** We now corrected it as follow: ‘’An interviewer-administered questionnaire was utilized to gather data. This questionnaire underwent translation into Bangla and was subsequently translated back into English with the help of experts. Before data collection began, all interviewers participated in a training session facilitated by the primary researcher to ensure consistency in their approach with different interviewees. The data collector initially posed a question to the responder, and after receiving an answer, the data collector repeated the response to confirm its accuracy.’’ In the revised text.

**Results**

The results section is scientifically robust and comprehensive, presenting valuable data on hand discomfort among professional motorcycle riders. However, minor revisions are recommended to improve clarity in variable definitions, reporting of model fit statistics, and presentation of tables. These enhancements will significantly increase the transparency, reproducibility, and scientific validity of the study.

**Reviewer’s comment:** The study reports that all 630 collected forms were complete and included in the final analysis, indicating high-quality data collection and the effective use of a digital tool (Google Forms) to prevent incomplete submissions. This is commendable and strengthens the credibility of the dataset. However, the authors should explicitly state how the built-in feature of Google Forms—preventing submission of incomplete responses—was integrated into the study design. This clarification would improve methodological transparency and assist other researchers in replicating the study.

**Reply to the reviewer’s comment:** We included the following statement in the revised text: ‘’We used the Google Forms® platform to gather responses to the survey as it is eco-friendly and it’s built in require option prevent incomplete submission.’’

**Reviewer’s comment:** Descriptive statistics (age, weight, height, BMI, education, marital status, income, riding experience, etc.) are well reported using appropriate measures (mean ± SD or percentages), allowing readers to clearly understand the study population. Nevertheless, for categorical variables such as education and marital status, it would be beneficial to also report absolute frequencies (number of participants), not just percentages. Providing counts enhances precision in data interpretation, especially for statistical models that depend on group sizes.

**Reply to the reviewer’s comment:** Thank you for your comment. To prevent duplication, we reported fewer component in the text and rest of the other are reported in the table 1.

**Reviewer’s comment:** The prevalence of hand discomfort in the right hand (58.3%) and left hand (51.3%) is clearly presented and includes valuable information on symptom frequency, severity, and work-related impact. Reporting daily frequency, discomfort intensity (mild, moderate, severe), and occupational interference demonstrates in-depth analysis and contributes to a better understanding of the disease burden. However, the authors should clarify whether these data were collected based on symptoms experienced in the past 7 days, as per the Cornell Hand Discomfort Questionnaire (CHDQ), to ensure readers understand the time frame of assessment.

**Reply to the reviewer’s comment:** Thank you for highlighting our unintentional mistake. We now addressed the time frame in the revised text.

**Reviewer’s comment:** Reporting the percentage of pain in each of the six hand regions (based on the CHDQ map) is highly informative and helps identify high-risk anatomical areas. For instance, region F (palm near the wrist) showed the highest prevalence of pain in the right hand, which may be associated with prolonged pressure on the handlebar. However, these findings would be strengthened by contextual interpretation highlighting the importance of motorcycle handlebar ergonomics.

**Reply to the reviewer’s comment:** Thank you for your comment, the following lines are added in the revised text: ‘’The highest prevalence of pain in the right hand was reported in area F (the palm and near the wrist), which may be linked to prolonged pressure on the handlebar while using the throttle. In contrast, a significant prevalence of pain was observed in area D of the left hand, likely associated with repetitive use of the clutch. These findings highlight the importance of motorcycle handlebar ergonomics.’’

**Reviewer’s comment:** The chi-square test results for associations between demographic/occupational factors and hand discomfort are well reported, including p-values for both hands, reflecting analytical rigor. Nevertheless, it would be beneficial to also report the chi-square statistic and degrees of freedom (df) so readers can independently assess the strength of the associations. Additionally, for variables showing no significant association (e.g., smoking, safety equipment use), a brief interpretative comment in the discussion—such as "the lack of protective effect from safety equipment may reflect actual inefficacy or improper use"—would add valuable context.

**Reply to the reviewer’s comment:** Thank you for your valuable comment. We now added the chi-square statistic and degrees of freedom (df) in table 2. We also included the following statement in the revised text: ''Additionally, this study found no association between hand syndrome and smoking, and the use of safety equipment may indicate that the lack of a protective effect reflects either actual ineffectiveness or improper use.''

**Reviewer’s comment:** The use of multivariable logistic regression and reporting of adjusted odds ratios (aOR) with 95% confidence intervals (CI) is a standard and robust approach for identifying predictors. This section is well structured and includes key variables such as age, BMI, education, riding experience, handlebar suitability, and bike-body fit. However, the dependent variable is not clearly defined (e.g., "presence of pain, ache, or discomfort in either hand in the past 7 days"). This definition should be explicitly stated in the text or in a footnote to Table 3 to avoid ambiguity.

**Reply to the reviewer’s comment:** Thank you for highlighting this issue. We now addressed this issue in the footnote of table 3.

**Reviewer’s comment:** The finding that individuals with normal BMI (18.5–22.9) have higher odds of hand discomfort compared to obese individuals may be misleading, as it contradicts expectations. The authors do not address the possibility that obese individuals in this sample may have lower exposure to hand stressors (e.g., fewer riding hours or different motorcycle types). Therefore, it is recommended that this unexpected finding be carefully interpreted in the discussion, avoiding causal claims. Additionally, BMI might have been more appropriately modeled as a continuous variable to assess linear trends.

**Reply to the reviewer’s comment:** We now addressed the following statement in the revised text: ‘’Obese individuals in this group may have lower exposure to hand stressors, such as fewer riding hours or different motorcycle types, resulting in the development of less pain syndrome.’’

**Reviewer’s comment:** The finding that riders with less than five years of experience have higher odds of hand discomfort is clinically significant and may reflect inadequate ergonomic adaptation or improper riding techniques. This result should be emphasized in the discussion as an opportunity for targeted training programs for new riders. It is also recommended to explore potential interaction effects between riding experience and other variables (e.g., motorcycle type or physical fitness) in future analyses.

**Reply to the reviewer’s comment:** The following statement has been included in the discussion section: ‘’Riders with less than five years of experience are more likely to experience hand discomfort, as indicated by this study. This finding is clinically significant and may suggest inadequate ergonomic adaptation or improper riding techniques. Policymakers should address this concern by focusing on proper ergonomic design and developing targeted training programs for new riders.’’

**Reviewer’s comment:** The finding that individuals whose motorcycle does not fit their body physique have over five times higher odds of hand discomfort is one of the strongest and most ergonomically logical results of the study. This should be highlighted as a key finding, and recommendations should be made in the discussion for ride-sharing platforms to implement policies regarding motorcycle selection or adjustment based on the rider’s height and weight.

**Reply to the reviewer’s comment:** Included the following statement in the revised text: ‘’Individuals whose motorcycles do not match their body physique have more than five times the likelihood of experiencing hand discomfort. It is strongly advised that ride-sharing platforms implement policies for motorcycle selection or adjustments based on the rider’s height and weight.’’ In the discussion section.

**Reviewer’s comment:** Although the methods section mentions the use of the Hosmer-Lemeshow test and VIF to assess model fit and multicollinearity, no information is provided in the results section about the actual model fit or VIF values. This is essential to assure readers that the model is valid and multicollinearity is not present. It is recommended to include this information in Table 3 or in the text, for example: *"The final model showed good fit (Hosmer-Lemeshow χ² = 6.21, p = 0.624), and all VIF values were below 3, indicating no multicollinearity."*

**Reply to the reviewer’s comment:** Thank you for your valuable comment. We now added the following statement in the data analysis section in the revised text as follow: ‘’The final model showed good fit (Hosmer-Lemeshow χ² = 4.765, p = 0.782 for the right hand and χ² = 10.68, p = 0.220 for the left hand), and all VIF values were below 3, indicating no multicollinearity.’’

**Reviewer’s comment:** In the regression section, there is no clear specification of the exact dependent variable (e.g., pain in either hand, right hand only, or any of the six regions). This ambiguity could lead to misinterpretation. It is recommended to explicitly define the dependent variable in the text or table footnote: *"Dependent variable: Presence of pain, ache, or discomfort in any of the six hand regions in the past 7 days, assessed by CHDQ."*

**Reply to the reviewer’s comment:** Addressed in the footnote of table 3 in the revised text.

**Reviewer’s comment:** Descriptive statistics for age, weight, height, and BMI are clearly reported using mean and SD, and key variables such as education level, marital status, and occupational status are appropriately included. However, the term "professional biker" requires clarification: does it refer exclusively to ride-sharing workers (e.g., Pathao, Uber), or does it include other categories of professional riders? This definition should be explicitly stated in the text. Additionally, data presentation could be more concise. Instead of listing every percentage in long sentences, key trends should be summarized, for example: *"The majority of participants (75.1%) were professional ride-sharing riders, and over two-thirds (68.1%) had less than five years of riding experience."* This approach improves readability and focuses attention on major demographic patterns.

**Reply to the reviewer’s comment:** We define the term ‘’professional biker’’ in study design section of the revised manuscript. We also re-phrase the sentences in the revised text.

**Reviewer’s comment:** The reporting of p-values for both hands reflects analytical precision, and a wide range of variables (age, BMI, education, income, riding experience, bike fit, etc.) were examined, demonstrating comprehensive analysis. However, clarity could be improved by grouping significant and non-significant variables. For example: *"Significant associations (p < 0.001) were found for age, BMI, education, income, and riding duration, while no significant association was observed with smoking or safety equipment use."* Furthermore, the repeated use of the phrase "pain, ache, or discomfort" after its initial definition is unnecessary. Once "biker’s hand syndrome" is defined, a consistent term such as "hand discomfort" should be used to improve readability.

**Reply to the reviewer’s comment:** Corrected in the revised text.

**Reviewer’s comment:** The use of adjusted odds ratios (aOR) with 95% CIs is appropriate and standard, and separate analyses for the right and left hands add depth to the findings. However, some interpretations are unclear or potentially misleading. For example, the statement "participants aged 31–40 exhibited a lower prevalence" is ambiguous without reference to the comparison group (e.g., those over 40). It should be rephrased as: *"Compared to participants over 40 years of age, those aged 31–40 had significantly lower odds of hand syndrome."* The unexpected finding that normal BMI is associated with higher odds requires careful interpretation in the discussion, as it may stem from misclassification, confounding, or statistical artifact. Additionally, strong associations related to poor bike fit (aOR = 5.136) and unsuitable handle grip (aOR = 2.110) should be highlighted as modifiable ergonomic factors and emphasized in the recommendations section.

**Reply to the reviewer’s comment:** Addressed in the revised text.

**Discussion**

This section has a solid foundation and reflects awareness of key methodological issues. With minor revisions—particularly in reducing overstatement, refining the discussion of limitations, and providing specific, actionable recommendations for future research—the conclusion will be more credible, coherent, and valuable to the scientific community.

**Reviewer’s comment:** The study appropriately highlights the use of face-to-face, interviewer-administered questionnaires as a strength, as this method likely reduced non-response and misclassification bias, particularly in a population with variable literacy levels. Additionally, the application of a two-stage cluster sampling technique enhances internal validity by minimizing selection bias. However, the claim that the study "encompasses the entire population" or could generalize to the "entire nation's populace" is inaccurate and misleading. The research was conducted only in two metropolitan areas and among professional ride-sharing riders, which limits national representativeness. To maintain scientific rigor, this should be revised to state that the sample is representative of urban professional riders in similar settings, rather than implying nationwide generalizability.

**Reply to the reviewer’s comment:** Thank you for bringing our unintentional mistake to our attention and for providing us the chance to enhance the quality of the manuscript. We now corrected the sentences as follow: ‘’Employing a two-stage cluster random sample method from two separate metropolitan areas improves result precision and reduces selection bias.  Including the entire nation's populace could increase the generalizability of the findings.’’

**Reviewer’s comment:** The suggestion that findings could be generalized to the entire nation’s population is problematic and should be revised. The study was limited to two major cities (Dhaka and Sylhet) and focused exclusively on professional motorcycle riders using ride-sharing platforms. Therefore, results may not apply to rural areas, smaller towns, or non-professional riders who may have different riding patterns, motorcycle types, and exposure levels. Generalizing beyond the studied population risks overinterpretation. Instead, the authors should clarify that findings are generalizable to similar urban, professional riding populations in comparable South Asian contexts, and future studies should explore variations across different regions.

**Reply to the reviewer’s comment:** Thank you for bringing our unintentional mistake to our attention and for providing us the chance to enhance the quality of the manuscript. We now corrected the sentences as follow: ‘’Employing a two-stage cluster random sample method from two separate metropolitan areas improves result precision and reduces selection bias. Including the entire nation's populace could increase the generalizability of the findings.’’

**Reviewer’s comment:** The authors appropriately recognize that the cross-sectional nature of the study prevents the establishment of causal relationships between variables. This is a crucial methodological limitation, and openly acknowledging it demonstrates scientific integrity. It allows readers to interpret the findings as associations rather than causal effects. This point should be reinforced in the discussion section, where the authors can recommend longitudinal or interventional studies to explore temporal relationships and potential causality, especially for modifiable risk factors such as bike fit and riding duration.

**Reply to the reviewer’s comment:** Thank you for your comment. We now addressed the following recommendations in the revised text: Future research should utilize longitudinal designs to assess the progression of symptoms or conduct intervention studies that focus on ergonomic modifications, such as handlebar design and glove usage, to evaluate their effects on hand pain syndrome. Furthermore, comparative studies involving non-professional riders or other transportation workers could offer extra information about occupational risk factors.

**Reviewer’s comment:** While the authors note that results may vary across urban or rural regions of Bangladesh, this limitation would benefit from a more detailed explanation. For instance, differences in road infrastructure, traffic congestion, motorcycle types, or work patterns could influence hand discomfort. The high traffic density and poor road conditions in Dhaka, compared to other cities, may amplify biomechanical stress on the hands. Clarifying these contextual factors would strengthen the argument and help readers understand why findings might not be transferable. Additionally, the exclusive focus on professional riders means results should not be extrapolated to casual or recreational riders without further evidence.

**Reply to the reviewer’s comment:** Thank you for your suggestion. The following statement has been addressed in the revised text: ‘’Variations in road infrastructure, traffic congestion, motorbike types, or work habits may influence hand pain. In Dhaka, the elevated traffic congestion and poor road conditions, relative to other cities, may augment biomechanical stress on the hands.’’

**Reviewer’s comment:** The authors rightly identify road pavement quality and traffic conditions as unmeasured factors that could influence hand syndrome. Uneven roads and frequent stop-and-go traffic increase hand vibration and grip force, both of which are known risk factors for nerve compression and musculoskeletal discomfort. However, this limitation should have been addressed earlier in the methods or results section to show proactive awareness. As it stands, mentioning it only in the limitations section appears reactive. Future studies should objectively measure or control for these environmental exposures—such as through GPS tracking, vibration sensors, or observational road assessments—to improve the accuracy of risk factor analysis.

**Reply to the reviewer’s comment:** The authors recognized this issue; however, due to logistical constraints, controlling those factors is not feasible at this time. Future studies should address these environmental factors to enhance the accuracy of risk factor analysis.

**Reviewer’s comment:** While the suggestion to study treatment needs and the chronic nature of hand syndrome is valid, it is too broad and lacks actionable direction. To be more impactful, future research recommendations should be specific, feasible, and directly linked to the study’s findings. For example: “Longitudinal studies with 6- to 12-month follow-up are needed to assess symptom progression,” or “Interventional trials should evaluate the effectiveness of ergonomic handlebar modifications or reduced daily riding hours.” Additionally, comparative studies involving non-professional riders or other occupational groups (e.g., auto-rickshaw drivers) could help isolate risk factors unique to motorcycle riding.

Need for logical integration and coherence across subsections: Currently, the subsections on strengths, limitations, and future recommendations are presented in a fragmented manner without a clear logical flow. To enhance coherence, each limitation should directly inform a corresponding recommendation. For example: “Due to the cross-sectional design, future longitudinal studies should examine symptom progression,” or “Given the unmeasured impact of road conditions, future research should include environmental assessments.” This structured approach would demonstrate a deeper engagement with the study’s implications and show that the authors are actively thinking about how to advance knowledge in this field. A well-integrated conclusion strengthens the manuscript’s overall impact.

**Reply to the reviewer’s comment:** Thank you for your insightful suggestion. We now upgraded our recommendation section as follow: ‘’Finally, the cross-sectional design of the current study suggests a need for future longitudinal research to examine symptom prognosis. Additionally, intervention studies focusing on ergonomic modifications, such as handlebar design and glove usage, should be conducted to assess their impact on hand pain syndrome. Furthermore, due to the unmeasured influence of road conditions, comparative studies that include non-professional riders or other transportation workers could help identify risk factors that are specific to motorcycle riding.’’ In the revised text.
